# Reactivity and Chemical Sintering of Carey Lea Silver Nanoparticles

**DOI:** 10.3390/nano9111525

**Published:** 2019-10-26

**Authors:** Sergey Vorobyev, Elena Vishnyakova, Maxim Likhatski, Alexander Romanchenko, Ivan Nemtsev, Yuri Mikhlin

**Affiliations:** 1 Federal Research Center Krasnoyarsk Scientific Center, Institute of Chemistry and Chemical Technology of the Siberian Branch of the Russian Academy of Sciences, Akademgorodok 50/24, 660036 Krasnoyarsk, Russia; likhatski@ya.ru (M.L.); romaas82@mail.ru (A.R.); yumikh@icct.ru (Y.M.); 2 Department of Chemistry and The Smalley-Curl Institute, Rice University, 6100 Main Street, Houston, TX 77005, USA; vishnyakovalena@mail.ru; 3 Federal Research Center Krasnoyarsk Science Center of the Siberian Branch of the Russian Academy of Sciences, Akademgorodok 50, 660036 Krasnoyarsk, Russia; ivan_nemtsev@mail.ru

**Keywords:** silver nanoparticles, Carey Lea colloid, citrate-derived capping, X-ray photoelectron spectroscopy, SEM, oxidation, sulfidation, sintering

## Abstract

Carey Lea silver hydrosol is a rare example of very concentrated colloidal solutions produced with citrate as only protective ligands, and prospective for a wide range of applications, whose properties have been insufficiently studied up to now. Herein, the reactivity of the immobilized silver nanoparticles toward oxidation, sulfidation, and sintering upon their interaction with hydrogen peroxide, sulfide ions, and chlorocomplexes of Au(III), Pd(II), and Pt(IV) was investigated using SEM and X-ray photoelectron spectroscopy (XPS). The reactions decreased the number of carboxylic groups of the citrate-derived capping and promoted coalescence of 7 nm Ag NPs into about 40 nm ones, excluding the interaction with hydrogen peroxide. The increased nanoparticles form loose submicrometer aggregates in the case of sulfide treatment, raspberry-like micrometer porous particles in the media containing Pd(II) chloride, and densely sintered particles in the reaction with inert H_2_PtCl_6_ complexes, probably via the formation of surface Ag-Pt alloys. The exposure of Ag NPs to HAuCl_4_ solution produced compact Ag films along with nanocrystals of Au metal and minor Ag and AgCl. The results are promising for chemical ambient temperature sintering and rendering silver-based nanomaterials, for example, for flexible electronics, catalysis, and other applications.

## 1. Introduction

Silver nanoparticles (Ag NPs) attract considerable attention from a wide range of fields such as catalysis, printed electronics, optics, biomedicine, and so forth, with the wet chemical synthesis is the main route for their manufacturing [1,2,3]. The very high-concentration Ag NPs sols are used as inks in the production of printed circuits and thin films with enhanced electric and thermal conductivity [3] and can provide large-scale synthesis of nanoparticles for other applications. However, high quantities of organic substances commonly added for aggregative stability of these sols should be thermally oxidized or removed in other ways in order to sinter or to reach required surface properties of silver nanoparticles. In the wet synthesis of noble metal nanoparticles, citrate-ions are widely employed as a cost-effective, green, and biocompatible reagent, which acts as complexing agent, reductant, and stabilizer of nanoparticles, and can be easily replaced with desirable surface ligands. In particular, the Turkevich method is a well-known technique for preparation of highly monodisperse gold hydrosols [4]. The citrate-assisted synthesis of silver nanoparticles is hampered by a low rate of reaction, which proceeds at elevated temperature or requires of an additional reductant [5]. In 1889, Carey Lea [6] has proposed a synthetic protocol involving silver nitrate, sodium citrate, and ferrous sulfate as reducing agent and yielding a stable, highly concentrated silver hydrosol at room temperature. The origin of the enhanced colloidal stability of the Carey Lea hydrosol is still unclear, since the studies devoted to these silver colloids and related immobilized particles are not numerous [7,8,9,10,11,12,13] and conducted with diluted sols. The low attention paid to this interesting system may be due to polydisperse Ag nanoparticles produced via the classical technique. It is generally assumed that the properties of the Ag NPs are due to adsorbed citrate anions [8,9,10], although it was recently demonstrated [14] that surface ligands are not citrate but the products of partial oxidation and decarboxylationof citrate, different on particles of distinct morphology.

Studies on reactivity have revealed some specific chemical properties of the silver nanoparticles including being stabilized by citrate and by its derivatives, although those remain far from being completely understood. In particular, Jolivet et al. [8] established that Carey Lea Ag NPs are charged negatively due to adsorbed citrate-ions and contain within their ligand shell Ag^+^ and Fe^2+^ ions, with the Ag^+^/citrate ratio being ~2. He et al. [15] have reported that citrate-stabilized Ag NPs initially react with H_2_O_2_ to form Ag^+^ and superoxide, which then re-form secondary Ag NPs with properties different from the initial citrate-capped nanoparticles. Li and Zhu [16] have established that in contrast to bulk metal, Ag NPs can react with HCl to give rise AgCl and H_2_. Mikhlin et al. [17] have shown that the citrate shell on the 10-nm-sized Ag NPs impedes the anodic oxidation of silver, preventing the formation of AgO. The interaction between nanosized silver and sulfur-bearing species has been found to result in the formation of separated particles or surface layers of Ag_2_S, Ag-Ag_2_S aggregates, core/shell Ag@Ag_2_S structures, and so on [18,19,20,21,22,23], depending on the reaction conditions. Composite nanostructures of silver with gold, and to a lesser extent, platinum and palladium are also widely discussed in the literature [24,25,26,27,28,29,30,31,32,33,34,35,36,37,38,39], including those obtained via a replacement reaction [28,34]. The reactions of metallic Ag with HAuCl_4_ solutions have been found to form alloys [25,26,28,29,30,31,32,33,34], Ag@Au core-shell structures [27,31,32], and hybrid particles [33]. Preparation of Ag–Pt and Ag–Pd nanocomposites ([36,37,38,39,40] and references therein) is of interest mainly as a method to modify activity and reduce the price of catalysts. At the same time, the reactivity of Carey Lea particles in reactions with noble metal chlorocomplexes has not been studied yet.

The aim of this study was to explore the chemical reactions of the Carey Lea nanoparticles immobilized on an inert support, occurring upon their interaction with various reagents (hydrogen peroxide, sulfide ions, tetrachloroauric acid, H_2_PtCl_6_, and H_2_PdCl_4_) in order to understand the mechanisms involved in the transformations of composition and morphology of Ag NPs. The results are expected to open new prospects for various applications of the unjustly neglected dense Carey Lea hydrosols and related species.

## 2. Materials and Methods

### 2.1. Materials

Silver nitrate (AgNO_3_), iron sulfate (FeSO_4_·7H_2_O), trisodium citrate (Na_3_Citrate·2H_2_O), and potassium nitrate (KNO_3_) were analytical grade and used as received. Deionized water (Millipore Milli-Q grade) was used to prepare all the solutions, to redisperse AgNPs, and to rinse the deposited samples.

### 2.2. Preparation of Silver Colloids and Films

In a typical procedure, 3.5 mL of solution of trisodium citrate (400 g/L) was mixed with 2.5 mL of a freshly prepared solution of ferrous sulfate (300 g/L of FeSO_4_∙7H_2_O), and the aqueous medium formed was added to 2.5 mL of 100 g/L AgNO_3_ solution under vigorous agitation. Black-bluish silver precipitate was produced, separated using centrifugation at 3000 rpm, the sediment was re-dispersed in 5 mL of deionized water, and then re-precipitated with 5 mL of 1 M KNO_3_ solution. The procedure was reproduced triply, and the last precipitate was suspended in deionized water, forming a dark brown hydrosol with the content of silver of 6 × 10^−2^ M. The films of immobilized nanoparticles were produced by drying a droplet of the Carey Lea sol on highly oriented pyrolytic graphite (HOPG) or other substrates, in particular copper foil, in air, and gentle washing the residue with water. HOPG was chosen as a substrate because of its chemical inertness, atomically smooth surface, and high conductivity, making it convenient for electron microscopy and XPS examination.

### 2.3. Chemical Treatment of Ag NPs

In this study, sulfidation of Carey Lea Ag NPs deposited on HOPG was performed by placing the sample under a bell together with a glass filled with 100 mL of 0.01 M Na_2_S aqueous solution for 30 min. Similar results were obtained also by utilizing a wet chemical treatment of the Ag NP films as described below.

The treatment of the immobilized Ag NPs via the wet chemical route was conducted by putting a ~20 μL drop of aqueous solutions of Na_2_S, H_2_O_2_, HAuCl_4_, H_2_PtCl_6_, or H_2_PdCl_4_ of a desired concentration onto the Ag film on HOPG (or other support), and kept for a predetermined time. After conditioning, the samples were cautiously rinsed with deionized water, dried in air, and examined with SEM and XPS.

### 2.4. Characterization

X-ray photoelectron spectra and X-ray-excited Ag M_4,5_NN Auger spectra were measured employing a SPECS instrument (SPECS Surface Nano Analysis GmbH, Berlin, Germany) quipped with a PHOIBOS 150 MCD 9 hemispherical analyzer at electron take-off angle 90° with the pass energy of 8 eV for high-resolution spectra and 20 eV for survey spectra using Mg K_α_ irradiation (1253.6 eV) of an X-ray dual-anode tube. The pressure in the analytical chamber was in the range of 10^−9^ mBar. The high-resolution spectra (C 1s, O 1s, Ag 3d, Ag M_4,5_NN, Au 4f, Pt 4f, Pd 5d, S 2p, and so forth) were fitted with Gaussian–Lorentzian peak profiles after subtraction of a Shirley-type background employing CasaXPS software (version 2.3.16, Casa Software, Teignmouth, UK).

Scanning electron microscopy (SEM) and energy dispersive X-ray analysis (EDX) were performed utilizing a Hitachi S5500 instrument (Tokyo, Japan) operated at acceleration voltage of 10 kV (in some cases, 30 kV). Defocusing the electron beam and other precautions were undertaken to avoid sintering the nanoparticles.

## 3. Results and Discussion

### 3.1. Intrinsic Ag Nanoparticles

**Figure 1:**
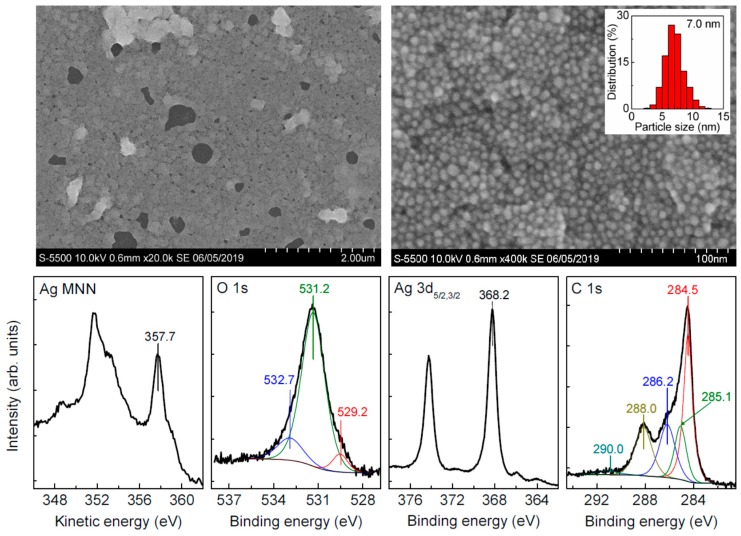
Typical scanning electron microscopy (SEM) images, particle size distribution, and X-ray photoelectron spectra (including X-ray-excited Ag M_45_NN Auger spectra) of unmodified Ag nanoparticle film deposited from Carey Lea hydrosol.

Figure 1 shows typical SEM images and X-ray photoelectron spectra of silver nanoparticles deposited from freshly prepared Carey Lea hydrosol. One can see that the Ag NPs are mainly rounded and uniform with a rather narrow size distribution centered at ~7 nm. The XPS Ag 3d spectrum of this sample exhibits the binding energy of Ag 3d_5/2_ line at 368.2 eV that is characteristic of Ag (0), in agreement with the kinetic energy of Ag M_5_N_45_N_45_ Auger peak at 357.7 eV [14]. The spectra are very narrow, indicating negligible contributions of oxidized silver species.

The C 1s spectrum is better fitted with four maxima, which can be ascribed to a contribution of underlying HOPG (284.5 eV), aliphatic carbon (285.1 eV) including some adventitious carbon, alcohol C–OH (286.2 eV), and carboxylic COO^−^ (288.0 eV) groups from capping ligands; a weak wide peak at 291 eV is due to a satellite from graphitic carbon. It should be stressed that the carboxylate/alcohol ratio derived from XPS is about 1.2, while it equals 3 in citrate (see [14] for more detail). This concurs with the O 1s spectrum, which can be fitted using the main peak at 531.3 eV attributable to hydroxide OH^−^ anions adsorbed at the film and oxygen atoms in carboxylate groups, the signal at ~533 eV from alcohol group, and possibly some chemisorbed water; the smaller maximum at 529.5 may originate from minor silver oxide Ag_2_O and, more likely, from Fe–O species remaining in the sample [14,41]. In general, the Ag NPs are more uniform in size and shape because of changes in the preparation receipt, whereas the XPS spectra agree well with those reported previously [14], and confirm that the ligands capping Ag NPs are actually the products of partial oxidation of citrate during the synthesis.

### 3.2. Sulfidation of Carey Lea Nanoparticles

**Figure 2:**
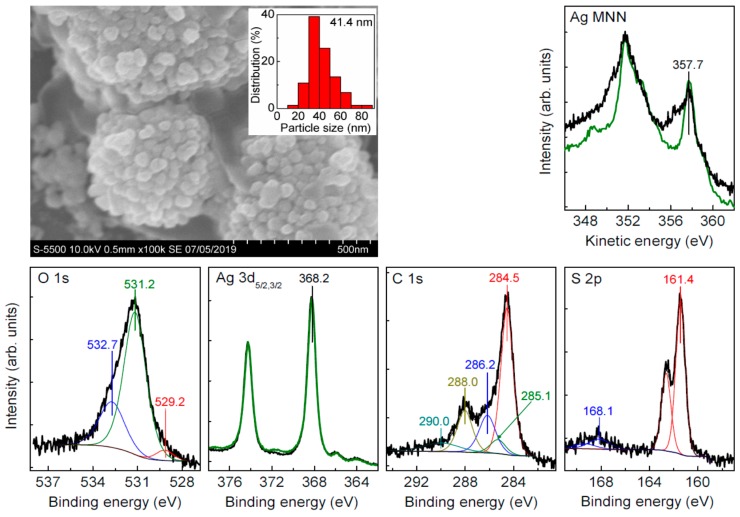
SEM image and X-ray photoelectron spectra of silver nanoparticles (Ag NPs) deposited from Carey Lea hydrosol and exposed with H_2_S via gas phase transfer for 30 min. For comparison, the Ag 3d and Ag M_5_NN spectra of the intrinsic nanoparticles (Figure 1) are shown as green lines.

We tried to conduct sulfidation of Ag NPs applying aqueous Na_2_S solutions, a portion of which was added to the Carey Lea hydrosol or placed onto the Ag NP film on a support, and alternatively via gas phase as described in Section 2.3. In the last case, more decent and reproducible results were obtained probably because the reactions of aqueous Ag^+^ ions and sulfide ions were absent. Typical SEM images acquired from the deposited Carey Lea particles after their sulfidation by gaseous H_2_S (Figure 2) show essentially aggregated Ag NPs, which are enlarged from 7 nm for the initial particles to 20–60 nm (the average size of 41 nm), and form rather loose aggregates of 300–800 nm in size. The XPS Ag 3d spectrum (Figure 2) insignificantly differed from the spectrum of initial Ag NPs, but a remarkable increase in the intensity at 356.5 eV for the Ag M_5_N_45_N_45_ Auger maximum indicates the formation of Ag(I)–S species. The major S 2p peak at 161.4 eV is typical for Ag_2_S [41], and a minor component with maximum at 168.1 eV is attributable to thiosulfate species as a product of sulfide oxidation in air. The Ag/S atomic ratio of 2.5, and higher proportion of elemental Ag in the spectra suggest a partial sulfidation of Ag NPs surfaces. Interestingly, the C 1s and O 1s spectra (Figure 2) only slightly changes, although the total content of carbon and oxygen decreases upon the reaction with sulfide species (Table 1), and the substitution of the protective citrate derivatives with sulfur and the formation of surface Ag_2_S appear to promote the Ag NP aggregation.

### 3.3. Oxidation of Silver Nanoparticles with Hydrogen Peroxide

**Figure 3:**
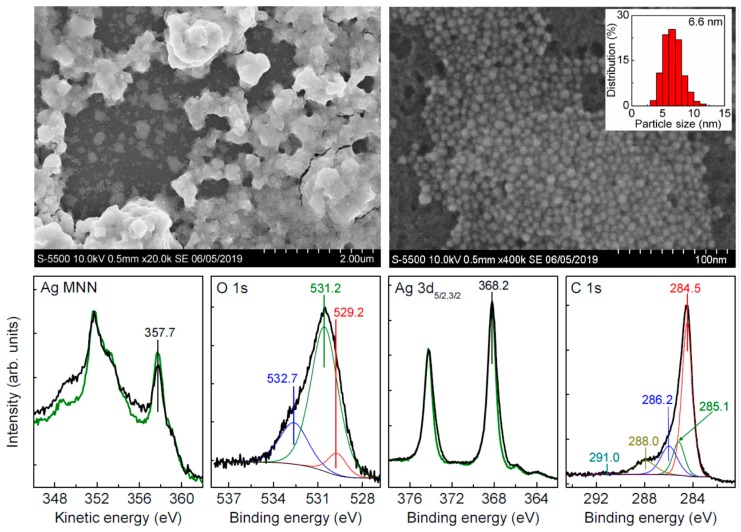
SEM images, particle size distribution, and photoelectron spectra of deposited Ag NPs and conditioned in 7 wt. % H_2_O_2_ solution for 5 min. For comparison, the Ag 3d and Ag M_5_NN spectra of intrinsic nanoparticles (Figure 1) are shown as green lines.

Typical SEM micrographs and photoelectron spectra of the Ag NP film treated with 7 wt. % H_2_O_2_ solution for 5 min are presented in Figure 3. No aggregation and sintering due to the treatment under those conditions were observed; variation of the concentration of hydrogen peroxide and the reaction time had an insignificant effect. The nanoparticles, however, became slightly smaller due to partial dissolution and possible re-precipitation of silver. The Ag 3d spectrum (Figure 3) is somewhat broadened at the low-energy side of the doublet maxima, and additional intensity appeared at the kinetic energies of 356.5 eV in the Ag M_5_N_45_N_45_ spectra, implying that up to 10% of Ag is presented as Ag(I) species. At the same time, the O 1s maximum shifts to lower binding energies due to the formation of OH^−^ and (or) O^2−^ species bonded to Ag atoms. The relative and total intensities of the signals from carboxylate groups decrease in comparison with that of alcohol both in the C 1s and O 1s spectra. These effects suggest further oxidation of citrate-derived ligands interrelated with partial oxidation and dissolution of Ag NPs via the reaction with hydrogen peroxide solutions.

### 3.4. Oxidation and Sintering of Ag NPs Film with HAuCl_4_ Solutions

**Figure 4:**
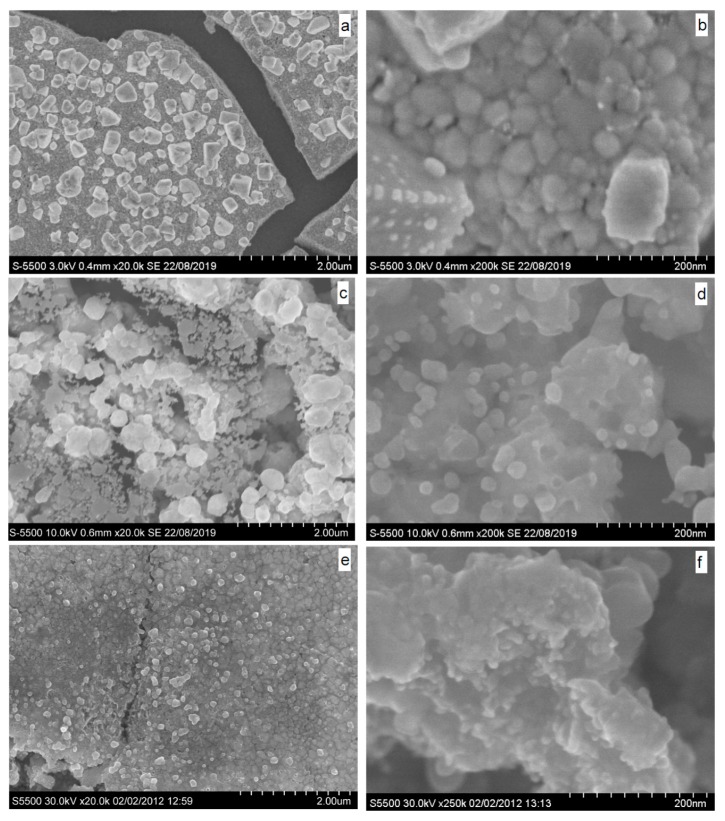
SEM images of silver films conditioned in (**a**,**b**) 0.2 mM HAuCl_4_ solution for 30 min and 4 mM HAuCl_4_ solution for 12 min (**c**,**d**) and 60 min (**e**,**f**).

**Figure 5:**
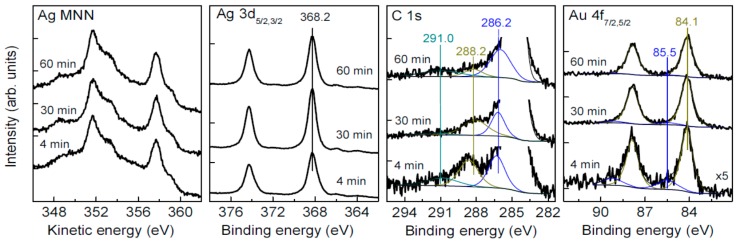
X-ray photoelectron spectra from silver films conditioned in 0.2 mM HAuCl_4_ aqueous solution for 4, 30, and 60 min.

SEM micrographs of Ag NPs deposited on HOPG and reacted with diluted (0.2 mM) and more concentrated 4 mM HAuCl_4_ solutions for 30 min and 12 min, respectively, are shown in Figure 4. Both nanoparticles increased to 20–60 nm and larger, densely sintered film islands and crystals are observed after the lower exposure to gold. At higher exposure, the monolithic material prevails in the film although some smaller “melted” particles occur too. X-ray photoelectron spectra from the Ag NPs reacted with 0.2 mM HAuCl_4_ solution for 4 min, 30 min, and 60 min are shown in Figure 5. The Ag 3d lines and Ag M_4,5_NN spectra are not broadened or shifted, with the Ag 3d_5/2_ maximum remaining at 368.2 eV. The gold signals appear and increase by a factor of ~3 with increasing the reaction time from 4 to 30 min (Table 2); the Au 4f spectra can be approximated by a component with Au 4f_7/2_ peak at 84.1 eV and a minor one at 85.5 eV, which should be attributed to Au(0) and Au(I) species, respectively. The C 1s spectra show a gradual decrease of carboxylate-to-alcohol ratio with time of reaction, and so the reduction of Au(III) and Au(I) species to elemental gold is coupled with the oxidative decarboxylation of the citrate-derived capping species. The surface concentrations of elements found using XPS (Table 2) revealed a decrease of oxygen content related to the oxidation of organic shell, and the increase of Au concentration that remains, however, less than silver content by at least 20 times.

**Figure 6:**
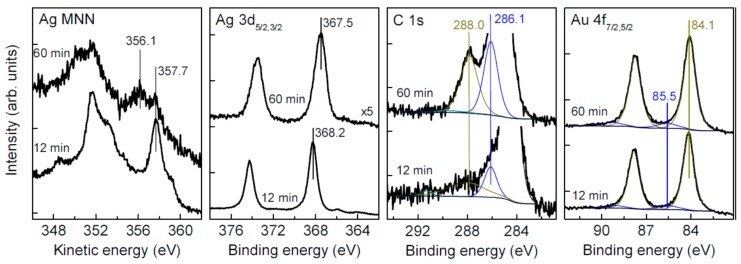
Ag NPs films treated with 4 mM HAuCl_4_ aqueous solution for 12 and 60 min.

Figure 6 shows SEM and XPS data (see also Table 2) for the Ag NPs reacted with 4 mM HAuCl_4_ solution. While the spectra of the sample prepared by the shorter treatment are not substantially different from those in Figure 5, despite a higher surface concentration of gold, the spectra are notably changed for the long reaction (60 min). In particular, Ag 3d_5/2_ maximum shifts to 367.5 eV, which is characteristic of Ag(I) species, and widens. The concentration of chloride is comparable with that of Ag, so the main part of surface silver seems to be in form of AgCl. The contents of Ag and Au are close, and the Au 4f spectrum contains, aside from the main peak of metallic gold at 84 eV, some contribution of Au(I) species, probably as gold chloride. It is interesting that the carboxylate-to-alcohol ratio in the C 1s spectra increases, probably due to adsorption of less oxidized citrate-derived species onto the surface of Au.

SEM images taken from the sample conditioned in 4 mM HAuCl_4_ solution for 60 min demonstrate strong sintering of the film composed both of smooth and rough areas, with a number of about 100 nm particles on top of the monolithic surface. EDX and data are rather close to the XPS analysis (Table 1) and show a uniform lateral distribution of Ag, Au, and Cl at the micrometer scale. These suggest that considerable part of elemental silver was converted to silver chloride and replaced by metallic gold rendering the film morphology.

### 3.5. Oxidation and Sintering of Ag NP Film with Solutions of H_2_PtCl_6_ and H_2_PdCl_4_

**Figure 7:**
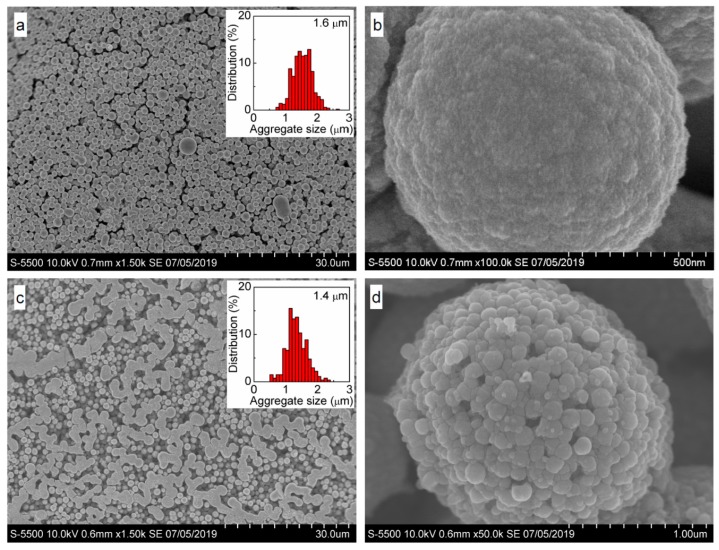
SEM images and particle size distribution of samples prepared via conditioning of deposited Carey Lea particles in 1 mM H_2_PtCl_6_ for 20 min (**a**,**b**), 0.33 mM H_2_PdCl_4_ solutions (**c**,**d**) for 40 min.

SEM images of the samples conditioned in aqueous H_2_PtCl_6_ and H_2_PdCl_4_ solutions (Figure 7) show that the Ag NPs film transformed into raspberry-like particles 1–2 μm in diameter. These micrometer spheres are composed of approximately 100 nm particles, which in turn consist of less than 10 nm nanoparticles, with both entities completely sintered after the treatment with H_2_PtCl_6_ solution. In the case of H_2_PdCl_4_ solution, the 100 nm particles are dense but are not closely agglomerated.

**Figure 8:**
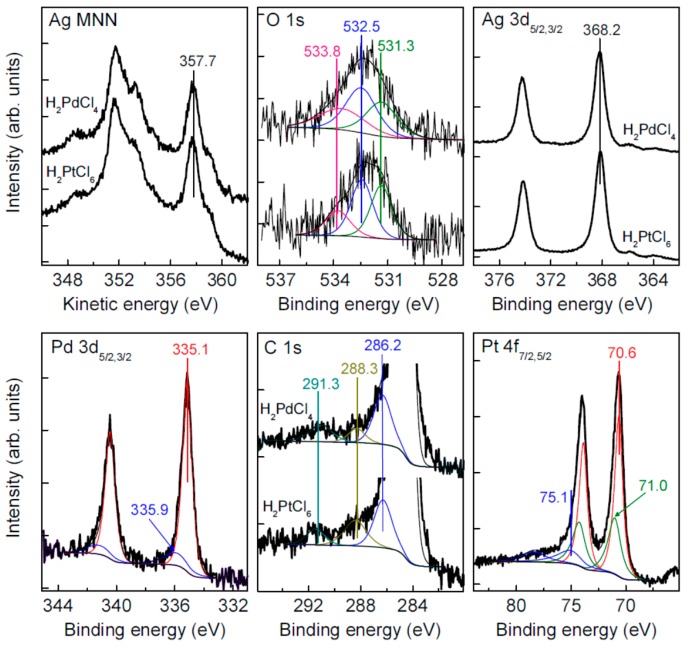
XPS spectra of silver films conditioned in H_2_PtCl_6_ solution for 20 min, and in H_2_PdCl_4_ solution for 40 min.

XPS analysis (Figure 8) found only elemental silver in these films; that is, the narrow Ag 3d_5/2_ peak at the binding energy of 368.2 eV and the Ag M_5_N_45_N_45_ maximum at the kinetic energy of 357.6 eV and no additional features as compared with the intrinsic Ag NPs (Figure 1). At the same time, the C 1s spectra exhibit a notable decrease of the relative concentration of carboxylate group (288 eV), with the carboxylate-to-alcohol ratio reduced to 0.3 after conditioning in the H_2_PdCl_4_ solution. The atomic ratios of Ag to Pt and Pd approach ~10, and the main lines of the Pt 4f_7/2_ and Pd 3d_5/2_ spectra at the binding energies of 70.7 and 335.2 eV, respectively, correspond to the elemental forms [38,42,43,44]. Moreover, the Pt 4f_7/2_ peak has even lower energy than bulk [42,43] metal or platinum nanoparticles [38] as a signature of electron density transfer from silver atoms. Such a phenomenon has been observed for Pt–Ag alloys [38] but the formation of core/shell structure or an interaction between Pt nanoparticles and silver substrate cannot be excluded too [45]. It is worth noting that the negative shifts of the photoelectron spectra were not observed here for gold and palladium.

## 4. On the Mechanisms Involved

**Figure 9:**
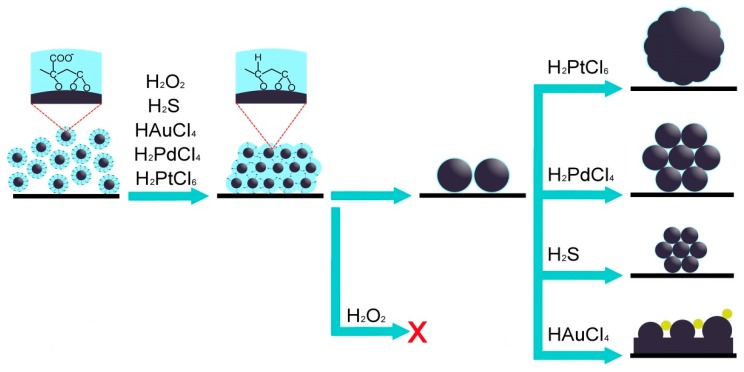
Scheme illustrating likely mechanisms of Ag NPs enlargement and sintering.

Oxidation or sulfidation of Carey Lea Ag nanoparticles is determined by the nature of reagents and the reaction conditions, and proceeds via oxidation or(and) substitution of protective capping ligands as the first stage. In fact, the capping ligands differ from citrate due to oxidation and partial decarboxylation of citrate during the synthesis [14], and further reaction carries on upon the interaction of immobilized Ag NPs with various oxidants (Figure 9). The Carey Lea Ag NPs seem to be less protected by the surface ligands and so more reactive than those capped with citrate or other adsorbates, especially than the particles deposited from concentrated sols (inks) containing large quantities of organic molecules. The decreasing number of surface anionic groups, and the surface charge, promoted coalescence of Ag NPs into larger silver particles, typically with the average size of 40 nm. However, we observed neither increased nanoparticles nor micrometer aggregates in the case of hydrogen peroxide, probably due to a higher concentration of aqueous Ag^+^ formed in the absence of chloride or sulfide ions [46]. The reaction of the Ag NPs with H_2_PtCl_6_ solutions results in effectively densely sintered micrometer raspberry spheres; this may be explained in terms of the formation of Ag–Pt alloys, probably because the interfacial reaction of Ag with inert Pt complexes is slow, allowing the solid-state transformation. In contrast, the Ag films are effectively sintered using Au(III) solutions due to a high rate of the cementation reaction, resulting in separated Au(0) crystals. At the high exposure, gold may completely substitute silver in the compact film. The rate of reaction of Ag nanoparticles with Pd(II) complexes and sulfide ions seem to be intermediary, yielding porous aggregates of 40 nm Ag NPs.

Although worthy of further investigation, the results shed new light onto the reactivity of silver nanoparticles, and demonstrate the prospects of the Carey Lea Ag NPs, whose morphology and composition can be easily modified by using oxidation (substitution) protective ligand capping, alteration of the surface layer, and chemical sintering of silver nanoparticles at room temperature.

## 5. Conclusions

We have studied reactivity of about 7 nm Ag nanoparticles protected with citrate-derived ligands immobilized from dense Carey Lea hydrosols towards sulfidation and oxidation with hydrogen peroxide, and the interaction with aqueous solutions of HAuCl_4_, H_2_PdCl_4_, and H_2_PtCl_6_ at room temperature. Sulfidation produced some surface silver sulfide and caused sintering of the Ag NPs into about 40 nm particles and their loose aggregates. Oxidation with hydrogen peroxide resulted in a decarboxylation of surface citrate-derived species and the formation of insignificant surface silver oxide, but no particle growth or aggregation was observed under the conditions applied. The interaction with aqueous chlorocomplexes of precious metals decreased the number of carboxylic groups of the citrate-derived capping and promoted coalescence of Ag NPs into dense 20–60 nm particles and then raspberry-like micrometer aggregates. The aggregates are densely sintered in the reaction with inert PtCl_6_^2−^ complexes, probably due to the formation of Ag-Pt alloys because of the slow interfacial reaction. A higher exposure of Ag NPs to HAuCl_4_ solution produced a compact Ag film along with some melted silver and gold entities.

## Figures and Tables

**Table 1:** Atomic concentrations found using XPS for the silver films before and after different chemical treatment.

Sample	C	Ag	O	S	Au	Pt	Pd	Fe	Cl
Initial silver film	46.2	30.3	22.0	–	–	–	–	1.5	–
+ H_2_O_2_ (7 wt. %)	51.1	26.8	21.4	–	–	–	–	0.7	–
+ H_2_S	36.9	32.1	18.5	12.5	–	–	–	–	–
+ HAuCl_4_ (4 mM, 12 min)	41.1	38.9	5.6	–	9.6	–	–	–	4.9
+ H_2_PtCl_6_ (1.0 mM, 20 min)	67.4	21.9	2.7	–	–	3.8	–	0.4	3.8
+ H_2_PdCl_4_ (0.33 mM, 40 min)	71.3	19.9	4.8	–	–	–	1.6	–	2.4

**Table 2:** XPS-derived surface concentrations for a series of silver films after different exposure to HAuCl_4_ solutions.

	Reaction Time (min)	Concentration (at. %)				
		C	O	Cl	Ag	Au
0.2 mM HAuCl_4_	4	34.4	14.2	10.4	38.3	0.5
	12	45.4	7.8	12.9	33.3	0.6
	30	54.4	8.5	3.6	31.7	1.2
	60	47.4	6.5	6.4	38.3	1.4
4 mM HAuCl_4_	12	41.1	5.6	4.9	38.9	9.6
	60	53.5	19.7	10.0	9.0	7.7
